# “He can send her to her parents”: The interaction between marriageability, gender and serious mental illness in rural Ethiopia

**DOI:** 10.1186/s12888-019-2290-5

**Published:** 2019-10-26

**Authors:** Maji Hailemariam, Senait Ghebrehiwet, Tithi Baul, Juliana L. Restivo, Teshome Shibre, David C. Henderson, Eshetu Girma, Abebaw Fekadu, Solomon Teferra, Charlotte Hanlon, Jennifer E. Johnson, Christina P. C. Borba

**Affiliations:** 1 Division of Public Health, College of Human Medicine, Michigan State University, East Lansing, MI USA; 2 College of Health Sciences, School of Medicine, Department of Psychiatry, Addis Ababa University, Addis Ababa, Ethiopia; 3 Department of Psychiatry, Boston Medical Center, Boston, MA USA; 4 Department of Global Health, Boston, MA USA; 5 Horizon Health Network, Fredericton, NB Canada; 6 Department of Psychiatry, Boston University School of Medicine, Boston, MA USA; 7 School of Public Health, Department of Preventive Medicine, Addis Ababa University, Addis Ababa, Ethiopia; 8 Center for Innovative Drug Development and Therapeutic Trials for Africa, College of Health Sciences, Addis Ababa University, Addis Ababa, Ethiopia; 9 Department of Global Health & Infection, Brighton and Sussex Medical School, Brighton, UK; 10 King’s College London, Institute of Psychiatry, Psychology and Neuroscience, Health Services and Population Research Department, Centre for Global Mental Health, London, UK

**Keywords:** Marriageability, Serious mental illness, Stigma, Discrimination

## Abstract

**Background:**

For women in most low- and middle-income countries, the diagnosis with serious mental illness (SMI) leads to stigma and challenges related to starting or maintaining marriages. The purpose of this qualitative study was to explore perspectives on marriage, divorce and family roles of women with SMI in rural Ethiopia.

**Methods:**

A qualitative study was conducted in a rural setting of Butajira, South Central Ethiopia. A total of 39 in-depth interviews were carried out with service users (*n* = 11), caregivers (*n* = 12), religious leaders (*n* = 6), health extension workers (*n* = 4), police officers (*n* = 2), teachers (*n* = 2) and government officials (n = 2). Data were analyzed using a thematic approach.

**Results:**

Three themes emerged. (1) Marriage and SMI: Chances of getting married for individuals with SMI in general was perceived to be lower: Individuals with SMI experienced various challenges including difficulty finding romantic partner, starting family and getting into a long-term relationship due to perceived dangerousness and the widespread stigma of mental illness. (2) Gendered experiences of marriageability: Compared to men, women with SMI experienced disproportionate levels of stigma which often continued after recovery. SMI affects marriageability for men with SMI, but mens' chances of finding a marital partner increases following treatment. For women in particular, impaired functioning negatively affects marriageability as ability to cook, care and clean was taken as the measure of suitability. (3) Acceptability of divorce and separation from a partner with SMI: Divorce or separation from a partner with SMI was considered mostly acceptable for men while women were mostly expected to stay married and care for a partner with SMI. For men, the transition from provider to dependent was often acceptable. However, women who fail to execute their domestic roles successfully were considered inept and would be sent back to their family of origin.

**Conclusion:**

Women with SMI or those married to partners with SMI are at greater disadvantage. Reducing vulnerabilities through stigma reduction efforts such as community outreach and mental health awareness raising programs might contribute for better social outcomes for women with SMI.

## Background

Serious mental illness (SMI) typically refers to psychotic disorders (eg. Schizophrenia and major affective disorders (eg. Bipolar disorder) that tend to be severe and enduring. Individuals with SMI experience substantially impaired functioning, interference of the illness with one or more major life activities, and worsening of other health and social outcomes [1]. The impact of illness also extends to families and caregivers who often shoulder the difficult role of caring for a family member with SMI [2]. As a result, both individuals with SMI and their caregivers are among the most vulnerable and disadvantaged segments of society.

In most low- and middle-income countries (LMICs), women with SMI experience multiple adversities due to untreated symptoms, functional impairment, limited access to treatment options, stigma, social exclusion and, more broadly, due to the sociocultural context that may disadvantage women in general [3, 4]. Women with SMI are at elevated risk of gender-based violence. For example, in a study from Nigeria, women with schizophrenia were at increased risk of intimate partner violence [5]. These gender disadvantages may translate into worse general health, economic, and social outcomes of SMI than men [3]. SMI is inextricably linked with poverty in most LMICs due to out of pocket healthcare financing and the long-term nature of the illness [6, 7]. Impaired functioning also leads to decreased productivity and reduced earning potential of families [8]. For these and other reasons, families of individuals with SMI fall into poverty due to precarious economic conditions.

In many communities, marriage is considered a union which earns the couple social approval and respect by the community. Being married is often taken as a marker of social status, a symbol of acceptance by the community and an ability to leverage vital social support [9]. In most rural parts of Sub-Saharan Africa, women’s roles are commonly limited to small-scale trading, farming, childbearing, cooking, cleaning, and caring [10]. SMI can interfere with women’s ability to fulfill these roles. However, failure to effectively execute these roles is often unwelcome and is a point of disapproval and social stigma. For example, in most rural parts of Ethiopia, being unable to cook and clean can be considered a reason for divorce.

Access to mental health treatment largely depends on the ability to mobilize the resources needed. Women with SMI may not access treatment due to lack of the financial means for seeking treatment or limited control over household resources necessary for accessing treatment, such as transportation or a means of payment [4]. Gender also plays a key role in making the decision about whether or not to initiate help-seeking or engage with treatment [4].

Health status affects marriage and marriageability worldwide [11]. Healthier people enjoy better chances of getting or staying married because they are considered attractive, and are believed to have better earning potential, mental well-being and perceived longevity [12]. Individuals with SMI may experience challenges entering into marriage and intimate relationships, especially when the condition is highly stigmatized. One common manifestation of this stigma is that individuals with SMI are not preferred partners in marriage and intimate relationships [13, 14].

Chances of marriageability could also be affected by mental illness of a family member. For example, even in high income countries such as Sweden, ‘stigma by association’ was reported to be common with family members of a person with SMI being distanced [15]. Similarly, studies from other LMICs (mainly India) indicate that not just the individual with SMI, but also their families and close relatives experience lower chances of marriageability [3, 16]. Nevertheless, literature focusing on marriageability, gender and family roles in Sub-Saharan Africa, especially in Ethiopia is limited. Previous work largely focused on mental health stigma in the domains of family stigma and self-stigma [17–19].

This study was carried out as part of a larger study designed to explore the sociocultural factors that contribute to the previously reported, significantly lower prevalence rates of SMI (schizophrenia) among women in Butajira, Ethiopia [20]. The aim of this specific study was to explore community perspectives/explanations towards marriage that may contribute to the observed SMI gender differences in this rural setting.

## Methods

### Study setting

The study was conducted in the South-central Ethiopia, in the Meskan district of the Gurage zone. Meskan is a field study site of Addis Ababa University [21] and is located 135kms South of Addis Ababa, the capital city of Ethiopia. The district has one psychiatric nurse-led outpatient psychiatric unit providing mental healthcare at Butajira general hospital. In most parts, help for mental health challenges is sought from traditional and religious healers [22]. Two-thirds of the population identify as followers of Islam [23]. Amharic is the *lingua franca* of the district.

Women in the district are uniquely disadvantaged. Majority of the women are under control of men, disempowered, with little to no say in making financial decisions and need permission from their husbands to seek healthcare [24]. Two-thirds of women in the district experience intimate partner violence in their lifetime [25]. Intimate partner violence rates were reported to be higher among literate rural women married to non-literate partners [26]. About 72% of married women in the district are not literate [27]. One in 25 women will have had her first child before the age of 18 [28].

### Study design

We used a qualitative design. A qualitative design was preferred because it allows deeper understanding of lived experiences, perspectives and other contextual factors around a complex social phenomenon [29–31]. In this study, we used this approach to aid contextual understanding of how culture interacted with marriage and marriageability, gender, family roles and expectations for individuals with SMI in this rural community.

### Sampling

We used a purposive sampling technique [32, 33] to identify participants for this study. A purposive list of participants representing diverse members of the community was generated by MH, CB and CH and was sent to a community gatekeeper. The participant groups in the list were identified based on their interaction with individuals with SMI in the community. Participants were recruited to the study by a community gatekeeper who had also worked as a data collector for a schizophrenia research project in the district for more than a decade [34]. This study enrolled a broad range of community stakeholders, including government officials, community health extension workers, police officers, teachers, religious leaders, traditional healers, as well as individuals with serious mental illness and their caregivers. The rationale for inclusion of these stakeholders was because of their repeated encounters with individuals with SMI and their families as part of their work. Participants with serious mental illness who were involved in this study were recruited from a cohort of individuals who had been followed as part of the Butajira schizophrenia research [35]. We selected participants who were in remission (for service users), over the age of 18 and were willing to participate in the study. We also included caregivers in the cohort who were taking care of an immediate family member with schizophrenia.

### Data collection

Data were collected from April–June, 2015. In collaboration with other researchers who have previous and ongoing research in the study site (CH, AF), open-ended topic guides were developed by one of the authors (CB). The topic guides covered 1) the impact of SMI on someone’s ability to get married 2) differential impact of SMI on marriageability of men versus women 3) impact of SMI on married life and 4) how it affects spousal and family support for men and women. Theoretical saturation, determined by repetition of the same stories, topics and themes guided the data collection process and the sample size. Amharic was the language of interview. Interviews were conducted by one female (MH; social work background) and one male interviewer (EG; public health background) matched to the sex of the interviewee. Both interviewers have qualitative research expertise and have previous experience conducting qualitative studies with the study population. Interviews were audio recorded, conducted face-to-face, and 60–90 min long. Confidentiality of the venue was considered. Study participants chose where to hold the interviews. Participants who appeared distressed during the interview or those who needed additional services were referred to the nearby health center and to other appropriate services available in the district.

### Data analysis

Interviews were conducted in Amharic and transcribed in the language of the interview verbatim. The interviews were translated to English by an experienced bilingual translator familiar with the study setting. Quality checks of the translation were done on a subset of randomly selected transcripts. The transcripts were imported into Open Code version 4.02, an open-source qualitative analysis software [36]. A codebook was derived from the data by MH, EG and CB. It was also updated with emerging themes. The codebook was used as a reference throughout the coding process. Two of the authors (MH and EG) coded the transcripts independently. A randomly selected 25% of the transcripts were double-coded by the same authors to establish consistency in coding. The two coders had over 85% agreement. Further analysis was also done by authors (CB, JR, TB and SG) to make sure that the identified themes were consistent with the data. Whenever discrepancies were noticed, the coders held a consensus meeting to discuss and resolve the disagreement. For concepts that were confusing, audio files of the interviews and interviewer field notes were used for clarification.

## Results

### Participant description

A total of 40 people were approached and one participant (a traditional healer) refused to participate. Thirty-nine people (16 females, 23 males) participated in the study. Eleven participants had a current diagnosis or past history of SMI. Sixteen participants reported that they had a family member with SMI. Study participants represented diverse educational, religious, age, professional, and social backgrounds (Table 1).

**Table 1:** Sociodemographic characteristics of study participants

	*N* (%) *N* = 39
Participants	
Service users	11 (28.2)
Caregivers	12 (30.8)
Religious leaders	6 (15.4)
Health extension workers	4 (10.3)
Police officers	2 (5.1)
Teachers	2 (5.1)
Government officials	2 (5.1)
Gender	
Male	23 (59.0)
Female	16 (41.0)
Age categories (years)	
18–24	5 (13.5)
25–34	13 (35.1)
35–44	6 (16.2)
45–54	8 (21.6)
55+	5 (13.5)
Marital Status	
Married	25 (67.6)
Not married	12 (32.4)
Educational attainment	
Cannot read/write	8 (21.6)
Primary school	13 (35.1)
Secondary school or greater	16 (43.2)
Religion	
Muslim	22 (62.9)
Orthodox	9 (25.7)
Catholic	2 (5.7)
Protestant	2 (5.7)

Three major themes emerged: (1) marriageability and SMI; (2) Gendered experiences of marriageability; and (3) acceptability of divorce and separation from a partner with SMI.

### Marriageability and SMI

Having SMI was reported to reduce chances of marriage for both men and women due to perceived dangerousness of individuals with SMI, mental health stigma, and the long-term nature of the illness in relation to those without SMI. The different groups of interviewees confirmed that people with SMI have generally narrower social circles, which often do not extend beyond family and close friendships. Stigma was mentioned as the primary limiting factor for social interaction of individuals with SMI.

*“People in the community don’t make friends with them [people with SMI]. They are usually lonely. No one wants to be with them. People are afraid to marry them. They will face problems until they recover and become healthy again”.* **Male service user, 26**

All of the participants agreed that marriage is taken as a marker of one’s place in the community. The decision to get married is taken very seriously and often involves parents of the couple, and SMI is seen as a major barrier to parental approval.

*There are many things people want to know before allowing marriage [even for couples without SMI]. Society wants to know about who he or she is, his or her ethnic group, who his or her parents are. It is a very big challenge. It is worse for a mentally ill person to marry in this society.* **Male caregiver, 45**

There also appeared to be general distrust towards people with SMI. Our respondents agreed that their society does not take people with history of serious mental illness (especially schizophrenia) seriously or consider them to be credible. Therefore, they were not considered trustworthy or good for any interpersonal relationship, including marriage.

*You cannot rely on what a mentally ill person says. They sometimes answer your questions correctly and they will answer incorrectly at other times.* **Female caregiver, 45**

Marriage between a person with history of mental illness and someone who does not have mental health problems was highly discouraged because the community believes that they “think differently.” Some participants endorsed the view that if someone is married to a person with mental health problems, the partner with mental illness might be violent and attack the other person.

*A mentally ill woman and a man who does not have mental health problems cannot get married and lead a happy life. They will not agree. They will think differently. They will not be able to live together because they are so different behaviorally.* **Male caregiver, 45**

Severity of illness was said to be the main factor lowering chances of marriageability among people with SMI. Individuals who have less severe psychotic episodes were believed to find a marital partner. Some participants remarked that marriage between two people with history of mental illness is easier than marriage with someone who doesn’t have mental illness.

*They will find marriage partners, if they are not completely crazy. They will find marriage partners who are [crazy] like them. A completely crazy person has greater chance of staying unmarried.* **Male traditional healer, 60**

This was also shared by another participant:

*A mentally ill woman might find a mentally ill man to marry her. A mentally ill man also might find a mentally ill woman to marry him*. **Male caregiver, 45**

In contrast, another group of participants believed that people with mental illness who received treatment have better chances of finding a marital partner albeit at lower rate than the general population.

*Most people with mental illness from this area have got married. There is this guy who receives medical treatment with us. He used to rip his clothes off of himself and walk around naked. He used to attack people and carry out arson attacks. He was not married [before he became mentally ill]. But, he married after they took him to Amanuel hospital and after he received [psychiatric treatment]. He is married to a beautiful wife and he has children now.* ***Female service user, 40***

### Gendered experiences of marriageability

All of the participants agreed that people with SMI experience challenges related to starting a family or maintaining long-term romantic relationships. However, women with SMI face especially difficult challenges due to inability to conform to gendered social and cultural roles and expectations surrounding them.

All of the interviewees confirmed that men’s superior role in the community is accepted unquestionably and is maintained through commonly established norms and values. Therefore, women’s chances of getting married or having a productive role in their communities are closely linked to their position in the society. All of the participants agreed that marriage is always initiated by men or family of the man. Women with SMI and their families have a passive role in marriage decisions.

Almost all of the participants agreed that men with SMI have better chances of getting married and starting a family than do women with SMI. A small minority of the participants reported that their chances of marriageability are similar. Men’s chances of finding a partner were also reported to increase following treatment and ability to provide for their family. However, this is not the case for women as the participants agreed that there is generally higher stigma around a woman’s mental illness.

*Our fathers say, women are, in nature, unable to express their feelings. They cannot express their feelings even when they are normal. And they think no one will marry them if they become mentally ill. They will not usually get married. Men can marry if they recover [from their mental illness] and can generate income and support themselves. It will not be difficult for men [to marry], if they recover and become normal.* **Male daily laborer, 26**

Arranged marriage is common in most parts of the district. Parents of the male usually get together, pick a potential wife for their son, and negotiate with her family, giving men a comparative advantage over women.

*Because the men start dressing themselves well and look good when they start taking the medication, they [women] will see that and marry them. Men are choosers. Women will not find husbands before friends and neighbors are asked about them [regardless of how well they groom themselves]. The illness is not recognizable in men*. **Female caregiver, 70**

Unlike men, women with SMI pass through a lot of scrutiny about their ability to cook, care, clean and maintain the household. Suitors often ask friends and neighbors about the ability of the woman to execute those responsibilities. Participants agreed that the recommendations from neighbors and friends can sometimes be a deal breaker for women. However, men with SMI are considered as eligible bachelors if they are not highly impaired.

Although marriageability seems to increase with treatment, past history of mental illness keeps haunting women more than men. Participants said that due to lack of access to treatment, it is common to see people with mental illness who strip their clothes and live on the streets. In such cases, it was mentioned that men’s nudity is tolerable and is normalized. Nevertheless, for a woman, having an experience of nudity during severe psychotic episodes further reduces her chances of getting married. Some of the participants interviewed said that the society is unforgiving towards a woman’s past history of mental illness. Getting married is difficult for women even after recovery.

*…but for the women, the community thinks that her illness will relapse anytime. The community will think about her previous situations, the situations where she stripped her clothes off herself and went out naked, even if she is healthy at the moment, because they are not educated and because they lack awareness….* ***Male teacher, 24***

Although not typical, some women conceal their mental illness and get married, however, husbands leave them when they find out that the women are mentally ill. On the other hand, having children and having less severe psychotic episodes was reported to reduce the likelihood of divorce:

*There are some people who have divorced [their wives because they are mentally ill]. Some people remarry them because they are the mothers of their children. People take care of a person whose illness hasn’t become severe.* **Male caregiver, 37**

Women’s marriageability is also linked to their ability to bear and raise children. Moreover, women are also expected to help with outdoor activities such as farming and gardening. A woman with mental illness is regarded as frail, hence, she is considered as the weakest partner who may not be of choice to anyone. Men who fail to fulfil their responsibility to provide receive pity and sympathy from the community. One of the participants who is a caregiver to her husband shared her view as follows: “*Being mentally ill is more disgraceful for women. It is very disgraceful. I wouldn’t leave him just because he is a man.”* Nevertheless, for women with SMI, inability to do household chores is considered as failure as a woman/wife, thus contributing to lower marriageability or subsequent divorce.

*If a man marries a woman, and she becomes mentally ill after that, if she doesn’t do anything in the house, if she doesn’t do any business, if she just sits and eats from what he brings home, he will leave her to marry somebody else. He might keep her in the house if she has children .*… *Women are more patient than men. A woman will wait patiently until the father of her children recovers from his illness and goes back to his work and becomes like his friends. She will take care of her husband. But a man will divorce his wife if she doesn’t do any business or cultivate their farm. He will leave her to marry another woman.* **Male service user, 38**

In addition to the widely prevalent stigma of mental illness towards people with mental illness, self-stigma is also one of the challenges for women to start a family. A woman with SMI stated her views as:

*Men might propose to me, but I will not be willing to marry because I am [mentally] ill. What if my illness relapses after I have got married and had children? I believe that my situation is unreliable.* **Female service user, 34**

Another female service user shared her experience as:

*…then I got married. I didn’t want to get married, because I am mentally ill. But they urged me to marry by asking me about what I am waiting for without marrying. Then I got married. But he [my husband] made me quit taking the medication, because he thought that I am crazy. It is my husband who made me quit [taking the medication]. The sickness in my head relapsed because of that.* ***Female service user, 18***

From the above experiences, it could be inferred that some of the women with SMI consider themselves less marriageable because of their diagnosis.

### Acceptability of divorce and separation from a marital partner with SMI

Almost all of the participants endorsed divorce or separation due to a wife’s mental illness as acceptable practice for men. However, they agreed that the society expects women to stay married regardless of the mental health status of their husbands. Participants said women should be caring and therefore it would raise a serious social disapproval if a wife decides to leave a husband with mental illness. They stated it is justified that some women can temporarily go away if their husband is abusive and violent towards them. The caring burden lies on the woman; men walking out of marriage is normal.

*Women are better. They will not leave their husbands. They will take care of their husbands and children. Women take responsibility for their family. They will not leave their husbands especially if they have children with them. But men leave their wives and marry another woman or they will send her to her parents.* **Health extension worker, 29**

The transition from provider to dependent due to onset or relapse of mental illness is mostly tolerated when it is a man because wives are expected to stay and care for their ill husbands. However, a woman’s impaired functioning often warrants divorce. Men are encouraged by their family, friends and neighbors to leave the marriage and remarry when the woman is mentally ill.

*When it is a woman, people will say, “Why doesn’t he leave her?”; “Why doesn’t he leave her?” ...“ Why is that he doesn’t leave her?” This is what they will say. There is again a greater challenge for women. His family will say, “Why doesn’t he leave her for somebody else?”…. If the man becomes ill, the problem will not be as difficult for him because the woman will take care of him. It is the man who will rush to divorce*. **Health extension worker, 27**

Separation due to mental illness often leaves women with limited access to household resources as described below.

*My husband left me. He said, “She is crazy”, “She will kill me”. The children are living with me. I left home. He got me out of the house. We had a small plot of land. The children vowed to take care of me and built a small house. I left the home because of that. We are living in that house. He is living in my matrimonial house with his wife. I planted vegetables and have done many other things in the compound. I had planted coffee, sugar-apple and papaya. If the children go there to get some crops, she [the second wife] will chase them with a stick. The children built a house and we are living in poverty.* **Female service user, 50**

The public attitude encouraged divorce for men when the wife has mental illness. However, women who are married to men with mental illness who considered divorce faced a more punitive societal attitude that often blames women for what went wrong. Below is a wife of a person with SMI:

*People will say he became ill because of me. He will die soon because he doesn’t eat food in other people’s houses, if he doesn’t have money…He has got a lot of problems. I don’t want to claim property or get money from him. What would people think of me*? *Had I gone to the police the moment he beat me, or when I was covered with blood, I would have expelled him from the house. We would have split our property…* **Female caregiver, 60**

Some of the wives caring for their husbands reported that they lived through serious physical and sexual violence especially when their husbands are having a psychotic episode.

*..Her husband [with SMI] started behaving very badly towards her. He started beating her. He was jealous. He didn’t want to see her outside the house. He didn’t want her to talk to anyone. I tried to act as a go-between and advise him…she finally filed for divorce and they separated.* ***Male community member, 50***

However, some of the women said they did not consider taking the case to court because they felt they needed to protect their husbands or feared the judgmental societal attitude towards women who pursue their rights. In some cases, the society blames women when a man develops mental illness after marriage. In the society, there is a belief that marital difficulties and other everyday stressors can cause mental illness and women are often blamed for it.

Most people agreed that men whose wives developed mental illness do not go the legal route of divorce because it entails splitting of the scarce family’s property. As a result, it is common for men to walk out of marriage or abandon their wives and marry another wife without having to undergo the formal divorce process.

*…Their love will fade. He may go see somebody else. This is when the woman is mentally ill…He can’t do that otherwise. He just can’t. Even if she is mentally ill, her rights are respected [by the law]. These days…women’s rights are better than men’s. The man can’t divorce his wife anytime. Because the properties will be equally shared on divorce.* **Male service user, 50**

## Discussion

This is the first published study of which we are aware that explores how gender interacts with marriageability and family roles of individuals with the diagnosis of SMI in a rural African setting. In this traditional community, the chances of getting married or starting a family was reported to decline following a diagnosis of SMI in both men and women. For women, the effect was reported to be more pronounced related to inability to discharge gendered roles in the personal and social realms of life. These findings could serve as an addition to the previous stigma literature in Ethiopia that focused on family stigma and internalized stigma of mental illness [17–19, 37].

In his seminal work, Ervin Goffman states that stigma is an attribute reducing the stigmatized into a tainted or discounted status [38]. Therefore, identification with a stigmatized condition can potentially lead to increased social disapproval and disqualification of those individuals from important social interactions (such as marriage). Our study participants mentioned that individuals with SMI were not considered trustworthy. Men’s eligibility for marriage was closely linked with their ability to provide. Women with SMI in particular are not considered as marriageable because they are not believed to have the ability to take care of household chores which are key markers of marriageability in rural communities. Thus, it was reported in our study findings that exclusion of individuals on the basis of their mental illness as ineligible for marriage was widely acceptable.

Similar to many parts of sub-Saharan Africa [39, 40], mental health stigma is a serious social problem in Ethiopia [17] due to perceived dangerousness and perceived supernatural causation of SMI [41]. A previous qualitative study from Ethiopia reported that the long-term nature of mental illness (chronicity) also contributes to higher levels of stigma [7]. High rates of health-related stigma negatively impact chances of getting married for individuals who are diagnosed with chronic conditions such as SMI. Hence, introducing interventions that limit their vulnerability and countering the stigmatizing attitudes and misconceptions with the correct information might improve social outcomes for individuals with SMI [42]. Even though study participants said the chances of getting married and having a productive role in the community increase with treatment more for men than women with SMI, access to treatment for women is important for their well-being and safety, and to reduce chances of abandonment. Efforts directed towards increasing access to mental healthcare should focus on ways to reach economically disadvantaged women, including women who are dependent on their husbands [4].

Some of the women with SMI involved in this study believed that they do not make a suitable marital partner because of their mental illness. This finding is consistent with what was reported by a previous facility-based quantitative study [18] that reported three-quarters of individuals with SMI endorsed at least one item in the internalized self-stigma of mental illness (ISMI) scale. In our study, self-stigma was also one of the barriers to getting married or starting a family, especially for women with SMI.

In traditional communities like many parts of rural Ethiopia, people may hold others responsible for causing mental illness [43]. For example, some of our participants agreed that women are to blame for men’s mental illness, especially when the onset of mental illness is after marriage. Raising mental health awareness and increasing mental health literacy, especially about the causes of mental illness, may help to combat this perception.

Previous studies have confirmed that violence against women at individual, family, group and community settings is widely tolerated in rural Ethiopia [44]. The country has one of the highest rates of violence against women, with up to 60% of women reporting domestic violence [45]. There is also a growing body of evidence suggesting that people with SMI might be targets of violent victimization in family and community settings [46]. Therefore, access to victim advocacy, protection of their rights, and gender empowerment with a focus towards women’s economic independence is crucial for both women with SMI and for women caring for partners with SMI.

Participants mentioned that historically, women in the study site didn’t share equal property after divorce. Therefore, it was culturally acceptable for men to end the marriage without any financial/economic implications. In recent years, there has been emphasis on women’s rights. Following that, court litigation of divorce led to splitting family properties to spouses. Raising awareness about property rights of women with SMI during divorce might be relevant to support their post-divorce maintenance.

This study has several strengths. It examined views from a wide range of stakeholders including individuals with SMI, families, healthcare professionals and traditional healers. Second, the study used a qualitative design which is an ideal tool to understand perceptions of a community towards SMI because it allows examining behaviors and beliefs as reported by the community. Finally, the study is novel and represents the first attempt to report perceptions of marriageability for individuals with SMI in a rural African setting.

The study also has some limitations. Due to logistical challenges, we were not able to go back to the participants to conduct member checking of the study results to receive their feedback on the findings. Second, both of our interviewers were urban and educated, which might have potentially contributed to social desirability bias. This may have resulted in more participants reporting positive views about marriage and family roles of individuals with SMI. To minimize the impact, however, we explained the purpose of the study and assured participants that there was no right or wrong answer. Finally, men with SMI were underrepresented in the study. This was mainly because of the original design of the study (that aimed to examine reasons for the reported lower prevalence of SMI in women).

## Conclusion

Outreach to rural women with SMI may be especially important given that these women may not have the resources or support of their families to attend treatment, and are at increased risk of abandonment by their marital partners, further increasing their vulnerability. Expanded access to mental health treatment might improve social outcomes of individuals with SMI. Reducing vulnerabilities through stigma reduction efforts such as community outreach and mental health awareness raising programs might contribute to better social outcomes for all individuals with SMI, but especially for women. Economic empowerment of women and victim advocacy might protect women with SMI and women who are caregivers of spouses with SMI.

## Data Availability

The used in the current study are not publicly available because the study is ongoing but are available from the corresponding author on reasonable request.

## Abbreviations

LMICs: Low-and Middle-Income Countries
NIMH: National Institute of Minority Health
SMI: Serious Mental Illness
